# Integrating Ecosystem Engineering and Food Web Ecology: Testing the Effect of Biogenic Reefs on the Food Web of a Soft-Bottom Intertidal Area

**DOI:** 10.1371/journal.pone.0140857

**Published:** 2015-10-23

**Authors:** Bart De Smet, Jérôme Fournier, Marleen De Troch, Magda Vincx, Jan Vanaverbeke

**Affiliations:** 1 Department of Biology, Marine Biology Research Group, Ghent University, Ghent, Belgium; 2 CNRS, UMR 7208 BOREA, Muséum National d’Histoire Naturelle, Paris, Cedex 05, France; 3 Station Marine de Dinard, USM 404, Muséum National d’Histoire Naturelle, Dinard, France; Università di Genova, ITALY

## Abstract

The potential of ecosystem engineers to modify the structure and dynamics of food webs has recently been hypothesised from a conceptual point of view. Empirical data on the integration of ecosystem engineers and food webs is however largely lacking. This paper investigates the hypothesised link based on a field sampling approach of intertidal biogenic aggregations created by the ecosystem engineer *Lanice conchilega* (Polychaeta, Terebellidae). The aggregations are known to have a considerable impact on the physical and biogeochemical characteristics of their environment and subsequently on the abundance and biomass of primary food sources and the macrofaunal (*i*.*e*. the macro-, hyper- and epibenthos) community. Therefore, we hypothesise that *L*. *conchilega* aggregations affect the structure, stability and isotopic niche of the consumer assemblage of a soft-bottom intertidal food web. Primary food sources and the bentho-pelagic consumer assemblage of a *L*. *conchilega* aggregation and a control area were sampled on two soft-bottom intertidal areas along the French coast and analysed for their stable isotopes. Despite the structural impacts of the ecosystem engineer on the associated macrofaunal community, the presence of *L*. *conchilega* aggregations only has a minor effect on the food web structure of soft-bottom intertidal areas. The isotopic niche width of the consumer communities of the *L*. *conchilega* aggregations and control areas are highly similar, implying that consumer taxa do not shift their diet when feeding in a *L*. *conchilega* aggregation. Besides, species packing and hence trophic redundancy were not affected, pointing to an unaltered stability of the food web in the presence of *L*. *conchilega*.

## Introduction

**Fig 1:**
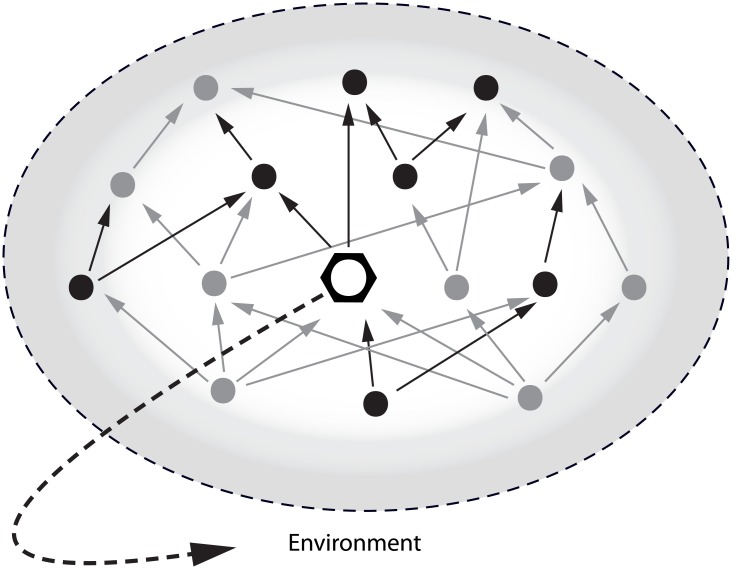
Conceptual model. Schematic representation of the expected impact of the ecosystem engineer *Lanice conchilega* on the structure of a soft-bottom intertidal food web. The engineer (hexagon), which is trophically coupled to the food web, affects the physical and biogeochemical characteristics of the environment (dotted arrow) and hence the base (primary producers) and higher trophic levels (macrofauna) of the food web. Consequently, the changes in the environment are expected to impact the overall structure of the food web (greyscale gradient). Nodes represent the primary producers and macrofaunal food web compartments, while arrows represent the trophic interactions, before (black) and after (grey) alteration by the engineer [based on 4].

Ecosystem engineers (species that contribute to the creation, modification or maintenance of the physical environment, which therefore have a crucial effect on other species [1]) and food webs are both well documented. The incorporation of non-trophic interactions in traditional food web studies is however only recently increasing [*e*.*g*. 2, 3], and up till now, the significance of the common and often influential process of ecosystem engineering on food web structure and dynamics remains largely unknown [4]. To get a more general understanding of interaction webs in nature, the integration of ecosystem engineering and food webs cannot be longer avoided [4]. Sanders *et al*. [4] recently presented a conceptual framework to integrate the largely independent research areas of ecosystem engineering and food webs. By structurally changing the abiotic environment, engineers can impact the structure of food webs either via node modulation (effect on the number of species that are present and their densities) or via link modulation (effect on the number and strength of trophic and non-trophic interactions among species) (Fig 1). The former also includes a subsequent change in links from the nodes to the rest of the food web [4]. Node and link modulation can operate on three non-exclusive engineering pathways; they can change the abiotic conditions (*e*.*g*. temperature and pH), the consumable abiotic conditions (*e*.*g*. nutrient leaching) and the non-trophic resources (*e*.*g*. competitor-free space). Via these pathways, the engineer might facilitate the addition of new producer species or alter the producer biomass and as such affect higher trophic levels [4]. The engineering pathways are believed to influence a food web at four possible levels: one trophic level, a food web compartment, a sub-set of species at different trophic levels or all species in the food web [4]. Moreover, if the engineer is trophically coupled to the food web (as a producer, consumer or decomposer), the net effect of the engineer on the food web will depend on a combination of engineering effects, trophic effects and positive or negative feedbacks to the engineer [4].

Despite the growing interest in the capacity of ecosystem engineers to modify the structure and dynamics of food webs, most studies dealing with this issue have a theoretical nature and empirical evidence is largely lacking [4–6]. So far, only a few recent studies have been looking at the link combining both research fields in the marine realm [*e*.*g*. 7, 8]. More evidence on how ecosystem engineers might affect food web structure and stability can be best provided by making use of an ecosystem engineer combining both autogenic (changing the environment via their own physical structures) and allogenic (changing the environment by transforming living or non-living materials from one physical state to another) engineering capacities [1]. Polychaete worms have been extensively studied regarding their ecosystem engineering capacities and the subsequent effects on soft-bottom communities [*e*.*g*. 9, 10, 11]. The terebellid polychaete *Lanice conchilega* is a prime example of an organism proven to be both an autogenic and allogenic ecosystem engineer [12, 13]. On the one hand, *L*. *conchilega* alters the biogeochemical properties of the environment by its bioirrigating activities [14, 15], while on the other hand it can appear as dense aggregations, creating biotic surface structures sometimes referred to as biogenic reefs [13], hence providing new habitats. In the presence of this engineering species, positive biodiversity and/or abundance and biomass effects have been reported on different size and/or ecological groups, ranging from primary producers (SPOM and MPB, [16, De Smet *et al*. unpublished]) to smaller meiofauna [*e*.*g*. 17, 18] and associated macrobenthos [*e*.*g*. 19] and hyperbenthos [16] up to (juvenile) (flat)fish and waders [*e*.*g*. 20, 21, 22].

While the structural and functional role of biogenic reefs has been well investigated, their impact on the food web structure has only been poorly considered. Following the conceptual framework of Sanders *et al*. [4], the habitats created by *L*. *conchilega* are expected to impact the overall food web structure via node modulation, since the tubeworm alters the primary producers’ abundance and biomass, and subsequently affect the nodes (*i*.*e*. species abundance and biomass) of the macrofaunal food web [16, De Smet *et al*. unpublished] (Fig 1; based on [4]). Therefore, we investigated the potential effect of biogenic *L*. *conchilega* aggregations on the structure of the macroscopic soft-bottom intertidal food web. We combined a classical approach (*e*.*g*. trophic position and functional groups) and a more integrative approach based on stable isotope analysis to study the food web structure. In ecological studies, the ^13^C/^12^C and ^15^N/^14^N stable isotope ratios are the most frequently used to infer primary food sources, trophic linkages and trophic position [23]. Generally, the quantitative information on both resource and habitat use provided by stable isotope analysis is utilised to define isotopic niche: an area (in δ-space) with isotopic values (δ-values) as coordinates [24], which should not be confused with an animal’s trophic niche [23]. Layman *et al*. [25] introduced a number of metrics that make use of stable isotope data to describe trophic structure ranging from individuals to entire communities and which can be used to compare trophic structure across systems or time periods. By implementing Bayesian statistics, Jackson *et al*. [26] provided improved estimates of the community metrics allowing for robust statistical comparison of isotopic niches of communities, both in space and time.

This study uses stable isotopes to investigate whether changes in the species composition in the presence of *L*. *conchilega* also causes changes in the structure of the food web. Moreover, to our knowledge, this is the first study that investigates the effect of an ecosystem engineer on the food web structure by using Bayesian Layman metrics. More specifically, we tested the hypotheses that (1) the ecosystem engineering activity of *L*. *conchilega* on its environment does not affect the isotope signatures of the primary food sources, and that (2) the altered abundance and biomass of primary producers and macrofauna in the presence of *L*. *conchilega* affects the structure, stability and isotopic niche of the consumer assemblage of a soft-bottom intertidal food web. Rather than focussing on one single ecosystem component, the entire bentho-pelagic consumer assemblage associated with the intertidal *L*. *conchilega* aggregation [16], was taken into account. To exceed the local scale, two different intertidal areas, representing different environmental settings along the French coast, were investigated.

## Material & Methods

### Study area and sampling design

Two soft-bottom intertidal areas located along the French side of the English Channel were sampled for primary food sources and consumer species. The bay of the Mont Saint-Michel (BMSM) is a large-scale intertidal sand flat located in the Normand-Breton Gulf (48°39.70’ N-01°37.41’ W; France); while Boulogne-sur-Mer (further referred to as Boulogne) is a small-scale beach along the northern part of the English Channel (50°44.01’N-01°35.15’E; Northern France). The locations were selected based on the presence of well-established intertidal *L*. *conchilega* aggregations.

At each location, the main primary food sources in the area (SPOM and MPB) and consumer species were sampled within a *L*. *conchilega* aggregation and a (control) area in the absence of any bioengineering species. The bathymetric level between the *L*. *conchilega* aggregations and their respective control areas was similar and the sampling areas were at least 300 m apart from each other. To include temporal variability in isotopic values, sampling took place in spring 2012 (from 7^th^ till 13^th^ of March in the BMSM and from 22^nd^ till 25^th^ of March in Boulogne) and was repeated in autumn 2012 (from 17^th^ till 21^st^ of September in BMSM and from 15^th^ till 18^th^ of October in Boulogne). Sampling was conducted in cooperation with the National Museum of National History (MNHN, Paris, France) and permitted by the ‘*Affaire Maritimes*’.

### Sampling of sources and consumers

Sediment particulate organic matter (SPOM) was collected by sampling the upper cm of the sediment during low tide. Upon return at the lab, artificial seawater was added to the sediment and following sonication and sieving (38 *μ*m), the supernatant was filtered onto precombusted (450°C for 2h) and pre-weighed Whatman GF/F filters (25 mm), temporarily stored frozen at -20°C and subsequently at -80°C until processing. Fresh microphytobenthos (*i*.*e*. benthic diatoms; MPB_diatom_) was collected by transferring the upper cm of the sediment to plastic boxes, covering the sediment with 100 x 150 mm Whatman lens cleaning tissue and cover slides, and putting the sediment under controlled light conditions enabling diatom migration. After about 2 days, diatoms were scraped off the cover slides, transferred to flacons with milli-Q water and centrifuged at 3000 rpm for 3 min. The diatom pellets were transferred to Sn capsules (30 mm Ø, Elemental Microanalysis UK), dried at 60°C and subsequently pinch closed and stored in Multi-well Microtitre plates under dry atmospheric conditions awaiting further analysis.

Macrobenthic invertebrates were collected with an inox corer (Ø 12 cm, 40 cm deep), sieved through a 1 mm circular mesh size and stored in a bucket with seawater. Upon return in the lab, animals were sorted, identified to the lowest possible taxonomic level, starved in seawater (24h) to allow evacuation of their gut contents and stored at -20°C before further treatment. In order to study the epi- and hyperbenthic communities, the lower water column (up to 40 cm) covering the sampling areas was sampled during daytime ebbing tide. Fish, shrimp and other epibenthic organisms were sampled with a beam trawl (2m long, 3m wide, 9 x 9 mm mesh size) equipped with a tickler-chain in the ground rope. Similarly, smaller animals living in the water layer close to the seabed (*i*.*e*. hyperbenthos; [27]) were collected with a hyperbenthic sledge consisting of a metal frame (100 x 40 cm) and equipped with two identical nets: a lower and an upper net (3 m long, 20 cm high at the mouth, 1x1 mm mesh size). The beam trawl and the hyperbenthic sledge were towed, either by a speedboat (Sillinger) in the BMSM or by foot in Boulogne, at a speed of 1 knot in the surf zone and parallel to the coastline for 100 m. Catches were sorted, identified to the lowest possible taxonomic level, starved in seawater (24h) to allow evacuation of their gut contents (only in case of smaller invertebrates), and stored at -20°C before further treatment. For each combination of location (BMSM *vs*. Boulogne), sampling area (*L*. *conchilega* aggregation *vs*. control area) and period (spring *vs*. autumn), 3 macrobenthic cores, 1 hyperbenthic catch and 1 epibenthic catch were collected.

### Sample preparation and stable isotope analysis

GF/F filters of SPOM and all collected consumer species were prepared for ^13^C and ^15^N stable isotope analysis. Frozen filters were thawed, dried overnight at 60°C and subsequently acidified by exposing them to HCl fumes (37%) in a dessicator to remove inorganic carbonates [28]. Filters were re-dried overnight at 60°C before being enclosed in Sn capsules (30 mm Ø, Elemental Microanalysis UK). In case of smaller invertebrates, such as polychaetes, amphipods and mysids, entire individuals were selected, whereas for fish and larger invertebrates, such as bivalves, crab and the brown shrimp *Crangon crangon*, only muscular tissue (dorsal fin, foot, cheliped and tail muscle tissue respectively) was prepared for analysis. Entire specimens and the dissected tissue samples were rinsed thoroughly with milli-Q water to avoid contamination and subsequently dried overnight at 60°C. Dried samples were grinded with a pestle and mortar, homogenized, weighted and encapsulated. The selection of the capsule depends on the need for acidification to remove carbonate traces [29], which was tested in advance. Invertebrates with calcareous structures such as shrimp, isopods and juvenile crab were transferred to Ag capsules (8 × 5 mm, Elemental Microanalysis UK) and acidified by adding dilute (10%) HCl drop-by-drop, until no more release of CO_2_ was observed [29]. Following acidification, samples were rinsed with milli-Q water, dried, pinch closed and stored in Multi-well Microtitre plates under dry atmospheric conditions until analysis. Carbonate-free tissue samples on the other hand, were encapsulated in Sn capsules (8 × 5 mm, Elemental Microanalysis UK), closed and immediately stored dry awaiting further analysis.

In total, 399 samples (both filters and animal tissue) were analysed at the UC Davis Stable Isotope Facility (University of California, USA) using a PDZ Europa ANCA-GSL elemental analyser, interfaced to a PDZ Europa 20–20 isotope ratio mass spectrometer (Sercon Ltd., Cheshire, UK). Stable isotope ratios are reported in the standard *δ* notation as units of parts per thousand (‰) relative to the international reference standards:

$$\delta X=\;\left[\left({\text{R}}_{\text{Sample}}/{\text{R}}_{\text{Standard}}\right)-1\right]\;\times \;10^{3}$$

where *X* is ^13^C or ^15^N and R is the corresponding ratio of ^13^C/^12^C or ^15^N/^14^N. Reference standards used were Vienna-Pee Dee Belemnite limestone (V-PDB) for carbon and atmospheric N_2_ for nitrogen. At least three replicates of SPOM and MPB_diatom_ were analysed for each combination of location, sampling area and period. In case of consumers, we strived to analyse at least three replicates per species, but for several taxa less replicates were available. In order to avoid pseudoreplication, every replicate equalled one single individual.

### Data analysis

This study incorporated two different sampling locations (BMSM and Boulogne) along the French coastal area. Analysis of the stable isotope data was performed for each of the locations separately since they are characterized by different environmental settings.

#### Primary food sources

Differences in the δ^13^C and δ^15^N isotope values of the primary food sources (SPOM and MPB_diatom_) between levels of the fixed factors sampling area (*L*. *conchilega* aggregation *versus* control) and period (spring *versus* autumn) were tested by a two-way ANOVA (Analysis of Variance). Significant interaction effects (*p* < 0.05) were further investigated by means of a TukeyHSD test. Prior to ANOVA, the assumptions of normality and homogeneity of variances were tested on untransformed data with Shapiro-Wilk tests and Levene tests respectively.

#### Consumers

The carbon and nitrogen isotope composition of consumer taxa co-occurring in both the *L*. *conchilega* aggregations and control areas were compared by plotting them in δ^13^C and δ^15^N biplots. Separate biplots were created for the locations and within biplots a distinction was made between periods. Taxa were assumed to have resembling δ^13^C and δ^15^N values in the *L*. *conchilega* and control areas if they fell within the 95% confidence interval (CI) encompassing the 1:1 correlation between *L*. *conchilega* aggregation and control isotope values.

To provide a detailed description of the entire food web structure, we used a classical approach and a more integrative approach. The classical approach includes trophic level determination and the clustering of taxa in trophic groups; the integrative approach consists of the estimation of community-wide metrics based on Bayesian statistics. Classification of consumers in groups of individuals with similar food uptake (δ^13^C) and trophic level (δ^15^N) was achieved by means of agglomerative hierarchical cluster analyses with group-average linking [30]. Clustering was performed for each of the combinations of location, sampling area and period separately and applied on a Euclidean distance resemblance matrix of normalised δ^15^N and δ^13^C isotope values of individual consumers. The clusters were examined for significant differences by similarity profile (SIMPROF) permutation tests [30]. Cluster analysis and SIMPROF test were performed using PRIMERv6 [30]. Furthermore, based on literature [*e*.*g*. 31] and the World Register of Marine Species (WoRMS; http://www.marinespecies.org/) consumers were classified into 8 functional groups: fish, predators, omnivore/predator/scavengers, omnivores, deposit feeders/facultative suspension feeders, suspension feeders, deposit feeders and herbivores.

A useful measure for the (dis)similarity of food web structure across different systems is the trophic position (TP) of consumers in a food web, which can be estimated based on the δ^15^N ratio [32, 33]:

$${\text{TP}}_{cons}=2+\frac{\left(\delta^{15}{\text{N}}_{cons}-\delta^{15}{\text{N}}_{base}\right)}{\Delta^{15}\text{N}}$$

where δ^15^N_cons_ and δ^15^N_base_ are the measured δ^15^N value of the consumer of interest and the δ^15^N value of the primary consumer used as the baseline respectively, 2 the TP of the primary consumer used as baseline, and Δ^15^N the trophic fractionation for δ^15^N per trophic level. When comparing a consumer’s TP between systems, the use of an appropriate trophic baseline is crucial [33, 34]. As a baseline, primary consumers are preferred to primary producers since they are spatially and temporarily less variable in their isotope values [33, 35]. The isopod *Lekanesphaera levii* (*L*. *conchilega* aggregation/spring; δ^15^N = 3.28‰) was selected as the trophic baseline for the food webs of the BMSM, while the amphipod *Gammarus sp*. (*L*. *conchilega* aggregation/spring and control/autumn; δ^15^N = 6.58‰) for Boulogne. In addition, the use of an appropriate trophic enrichment factor (Δ^15^N) is important because consumers are typically enriched in their C and, mainly, N ratios relative to their prey. The generally accepted Δ^15^N of 3.4 ‰ was used because it was proven to be a robust and widely applicable assumption when applied to entire food webs [33, 36].

The structure and niche widths of the food webs were investigated by calculating 6 descriptive community-wide metrics based on stable isotope data. The metrics were originally proposed by Layman *et al*. [25] and reformulated in a Bayesian framework by Jackson *et al*. [26]. Trophic diversity within the community is reflected by the total extent of spacing within δ ^13^C–δ ^15^N biplot space and measured by the first four metrics: *δ* ^*15*^ *N range* (NR; representation of the vertical food web structure), *δ* ^*13*^ *C range* (CR; representation of diversity at the base of the food web), *total area* of the convex hull encompassing the data (TA; representation of the niche space occupied) and *mean distance to centroid* (CD; representation of the average trophic diversity within the food web). The extent of trophic redundancy (the relative position of taxa to each other within niche space) is measured by metrics five and six: *mean nearest neighbour distance* (MNND) and *standard deviation of the nearest neighbour distance* (SDNND). These metrics were calculated based on standard ellipses [37] and Bayesian methods resulting in improved estimates, including their uncertainty [26]. However, because the TA is highly sensitive to sample size and hence impedes comparison between communities with unequal sample sizes, Bayesian standard ellipse area (SEA_B_) was used. Standard ellipses are not sensitive to sample size because they generally contain about 40% of the data. Nevertheless, for small sample sizes (n < 30) the tendency towards underestimating the SEA remains. Therefore, a small sample-size corrected standard ellipse (SEA_c_), insensitive to sample size [26], was calculated.

All univariate analyses were run in R (Version 3.1.2). The calculation of the Bayesian Layman’s metrics and standard ellipse areas for the different communities was done using SIBER (Stable Isotope Bayesian Ellipses in R; [26]).

## Results

### Primary Food Sources

**Fig 2:**
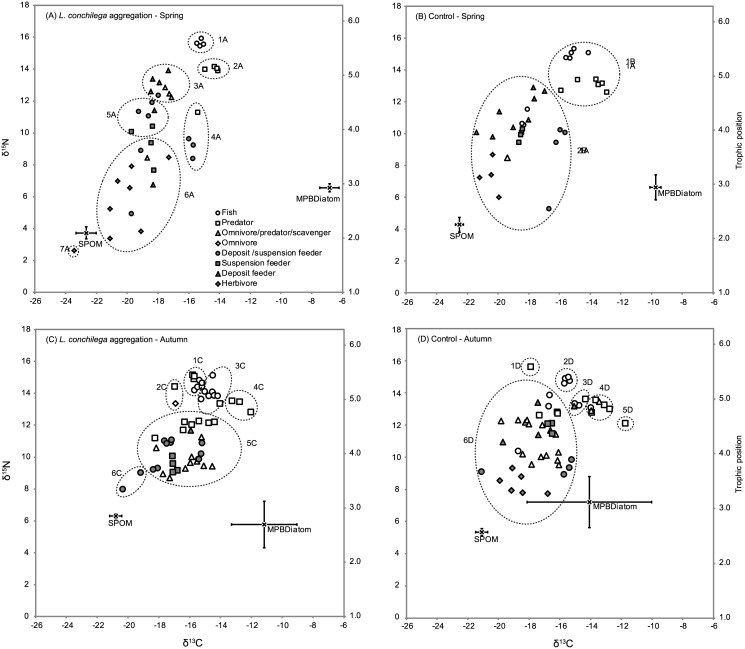
Stable isotope biplot for the bay of the Mont Saint-Michel (BMSM). Biplot of δ^13^C and δ^15^N isotope values (‰) of the primary food sources (mean ± SD) and of individuals of consumer taxa of the soft bottom intertidal area of the bay of the Mont Saint-Michel for different combinations of sampling area (*L*. *conchilega* aggregation *vs*. control) and period (spring *vs*. autumn). (A) = *L*. *conchilega* aggregation—spring; (B) = control—spring; (C) = *L*. *conchilega* aggregation—autumn; (D) = control—autumn. The trophic position (TP) of the consumer taxa, based on the isopod *Lekanesphaera levii* as a baseline, are displayed on the right of the biplot. Symbols/shading represent the 8 different functional groups (fish, predator, omnivore/predator/scavenger, omnivore, deposit/facultative suspension feeder, suspension feeder, deposit feeder, herbivore). Dashed ellipses represent the trophic groups delineated based on agglomerative hierarchical cluster analyses and similarity profile (SIMPROF) permutation tests. The mean δ^13^C and δ^15^N values (±SD) of the clusters, as well as their taxonomic composition are listed in S1 Appendix.

In the BMSM, MPB_diatom_ (ranging from -14.08 ± 4.07‰ to -6.84 ± 0.63‰) showed a more enriched δ^13^C value than SPOM (ranging from -22.66 ± 0.66‰ to -20.79 ± 0.39‰) for all sampling areas and periods (Fig 2). The δ^13^C value of both SPOM and MPB_diatom_ was affected by the factor period: δ^13^C of SPOM was lower in spring than in autumn, while δ^13^C of MPB_diatom_ was higher in spring than in autumn (Table 1). δ^15^N values in the BMSM ranged from 3.72 ± 0.38‰ (SPOM in the *L*. *conchilega* area during spring) to 7.21 ± 1.60‰ (MPB_diatom_ in the control area during autumn) (Fig 2). No differences in δ^15^N of MPB_diatom_ could be detected, while the δ^15^N value for SPOM differed among the sampling area x period interaction (Table 1). However, only in autumn the δ^15^N value of SPOM was significantly higher in the *L*. *conchilega* aggregation compared to the control area (Table 1).

**Table 1:** Two-way Analysis of Variance (ANOVA) and pair-wise Tukey HSD test results for the carbon (δ13C) and nitrogen (δ15N) stable isotope values of the primary food sources (SPOM and MPBdiatom). Sampling area (*L*. *conchilega* aggregation vs. control) and period (spring vs. autumn) were fixed factors. Analyses were performed on untransformed stable isotope data and run separately for the bay of the Mont Saint-Michel (BMSM) and Boulogne-Sur-Mer (Boulogne). Pair-wise Tukey HSD test results were shown for significant sampling area x period interactions. In case of significant differences (*p* < 0.05) *p* values are in bold.

		Main test												Pair-wise test	
		Sampling area				Period				Sampling area x Period				Spring	Autumn
Location	Source	SS	df	F-value	*p* value	SS	df	F-value	*p* value	SS	df	F-value	*p* value	*p* adj	*p* adj
**BMSM**	δ^13^C SPOM	0.14	1	0.6839	0.4322	3.04	1	15.0586	**0.0047**	0.15	1	0.7305	0.4176	―	―
	δ^15^N SPOM	1.38	1	12.564	**0.0076**	1.69	1	15.318	**0.0045**	1.73	1	15.749	**0.0041**	0.2418	**0.0310**
	δ^13^C MPB_diatom_	14.55	1	3.3218	0.0956	32.30	1	7.3747	**0.0201**	0.00	1	0.0001	0.9943	―	―
	δ^15^N MPB_diatom_	3.58	1	2.9043	0.1164	0.60	1	0.4891	0.4989	1.76	1	1.4264	0.2575	―	―
**Boulogne**	δ^13^C SPOM	2.76	1	17.715	**0.0030**	17.20	1	110.39	**<0.0001**	2.65	1	17.009	**0.0033**	0.4186	**0.0126**
	δ^15^N SPOM	0.001	1	0.002	0.9655	0.30	1	0.568	0.4726	0.26	1	0.4827	0.5069	―	―
	δ^13^C MPB_diatom_	30.20	1	4.2622	0.0659	71.55	1	10.097	**0.0099**	55.33	1	7.8073	**0.0190**	0.2924	0.2289
	δ^15^N MPB_diatom_	2.43	1	3.828	0.0821	1.06	1	1.6756	0.2277	0.03	1	0.0517	0.8253	―	―

**Fig 3:**
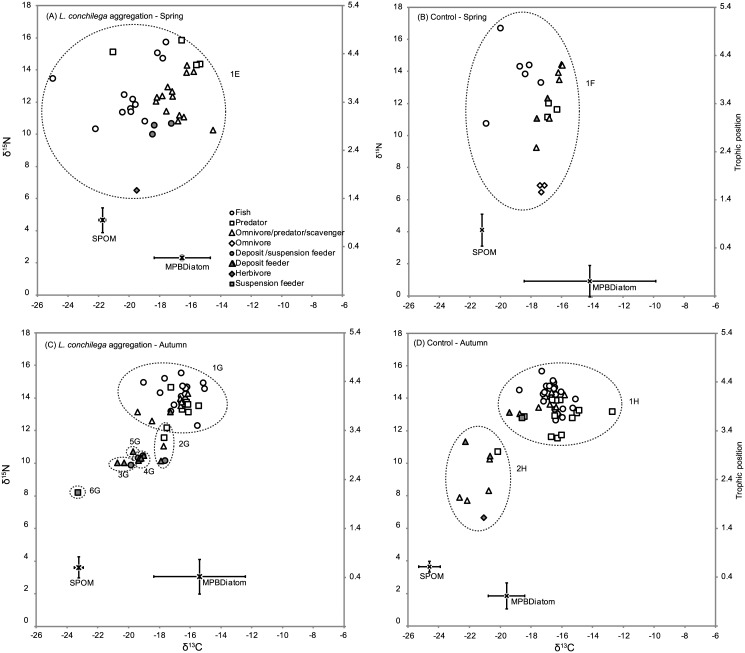
Stable isotope biplot for Boulogne. Biplot of δ^13^C and δ^15^N isotope values (‰) of the primary food sources (mean ± SD) and of individuals of consumer taxa of the soft bottom intertidal area of Boulogne-sur-Mer for different combinations of sampling area (*L*. *conchilega* aggregation *vs*. control) and period (spring *vs*. autumn). (A) = *L*. *conchilega* aggregation—spring; (B) = control—spring; (C) = *L*. *conchilega* aggregation—autumn; (D) = control—autumn. The trophic position (TP) of the consumer taxa, based on the amphipod *Gammarus sp*. as a baseline, are displayed on the right of the biplot. Symbols/shading represent the 8 different functional groups (fish, predator, omnivore/predator/scavenger, omnivore, deposit/facultative suspension feeder, suspension feeder, deposit feeder, herbivore). Dashed ellipses represent the trophic groups delineated based on agglomerative hierarchical cluster analyses and similarity profile (SIMPROF) permutation tests. The mean δ^13^C and δ^15^N values (±SD) of the clusters, as well as their taxonomic composition are listed in S2 Appendix.

In Boulogne, MPB_diatom_ (ranging from -19.58 ± 1.18‰ to -12.67 ± 3.97‰) showed a more enriched δ^13^C value than SPOM (ranging from -24.60 ± 0.70‰ to -21.21 ± 0.10‰) for all sampling areas and periods (Fig 3). The δ^13^C value of both SPOM and MPB_diatom_ was affected by the interaction of sampling area x period (Table 1). Pair-wise tests showed that the δ^13^C value of SPOM in the *L*. *conchilega* aggregation was significantly higher than in the control area but only in autumn (Table 1). No significant pair-wise differences could be detected for MPB_diatom_. δ^15^N values in Boulogne range from 0.91 ± 0.99‰ (MPB_diatom_ in the control area during spring) to 4.66 ± 0.77‰ (SPOM in the *L*. *conchilega* aggregation during spring (Fig 3). Nor for SPOM, neither for MPB_diatom_ differences in δ^15^N for the factors sampling area and period could be detected (Table 1).

### Consumers

**Fig 4:**
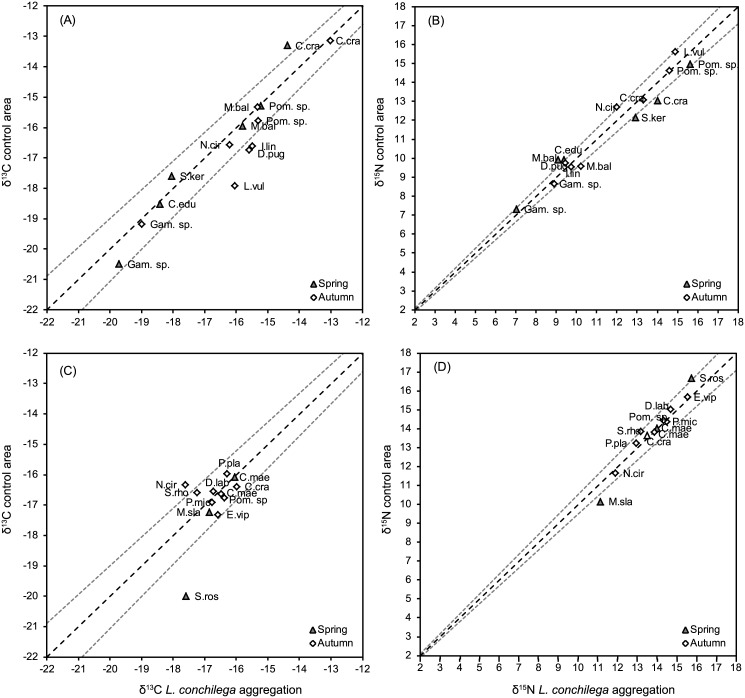
Comparison of consumer isotope values between the *L*. *conchilega* aggregations and the control areas. Separate plots for δ^13^C and δ^15^N isotope values per location are displayed: δ^13^C values in the BMSM (A) and Boulogne (C); δ^15^N values in the BMSM (B) and Boulogne (D). The central dashed line represents a 1:1 correlation between isotope values in the *L*. *conchilega* aggregations *vs*. control areas. A 95% confidence interval is represented by the outer dashed lines. Consumer taxa within the 95% confidence interval are not significantly different between the *L*. *conchilega* aggregation and the control area. Only consumer taxa which were collected both in the aggregations and control areas were taken into account. Species abbreviations: C. mae = *Carcinus maenas*; C. edu = *Cerastoderma edule*; C. cra = *Crangon crangon*; D. lab = *Dicentrarchus labrax*; D. pug = *Diogenes pugilator*; E. vip = *Echiichthys vipera*; Gam. sp. = *Gammarus* sp.; I. lin = *Idotea linearis*; L. vul = *Loligo vulgaris*; M. bal = *Macoma balthica*; M. sla = *Mesopodopsis slabberi*; N. cir = *Nephtys cirrosa*; P. pla = *Pleuronectes platessa*; P. mic = *Pomatoschistus microps*; Pom. sp. = *Pomatoschistus* sp.; S. ker = *Schistomysis kervillei*; S. rho = *Scopthalmus rhombus*; S. ros = *Syngnathus rostellatus*.

In total, 346 organisms belonging to 71 taxa were collected and analysed for their stable isotope values. 188 organisms (46 taxa) inhabiting the BMSM were analysed, of which 97 organisms (36 taxa) were collected in the *L*. *conchilega* aggregation and 91 organisms (38 taxa) in the control area; while for Boulogne 158 organisms (42 taxa) were analysed, of which 82 organisms (34 taxa) were collected in the *L*. *conchilega* aggregation and 76 organisms (30 taxa) in the control area. The majority of organisms in the BMSM were crustaceans (52.1%) and fish (18.6%), as was the case for Boulogne (40.5% en 39.9% resp.) (Tables 2 and 3). Both for the BMSM (Fig 2) and Boulogne (Fig 3), the most depleted δ^13^C values were found in the *L*. *conchilega* aggregations during spring (BMSM: *L*. *levii* = -23.45‰; Boulogne: *Pleuronectes platessa* = -24.99‰) and the most enriched δ^13^C values in the control area during autumn (BMSM: *C*. *crangon* = -11.75‰; Boulogne: *Liocarcinus* sp. = -12.71‰). Regarding δ^15^N, the most depleted (*L*. *levii* = 2.62‰) and most enriched (*Pomatoschistus* sp. = 15.93‰) values for the BMSM were found in the *L*. *conchilega* aggregation during spring, while for Boulogne the most depleted (*C*. *crangon* juvenile = 6.46‰) and most enriched (*Syngnathus rostellatus* = 16.68‰) values were found in the control area during autumn. In general, most taxa fell within a 1:1 correlation of isotope values between a *L*. *conchilega* aggregation and control area, *i*.*e*. their δ^13^C and δ^15^N values in the *L*. *conchilega* aggregation resembled those of the control areas (Fig 4). However, isotope values for some taxa were enriched (on the right side of the 95% CI) or depleted (on the left side of the 95% CI) in the *L*. *conchilega* aggregation compared to the control area. In the BMSM during autumn, enriched δ^13^C values in the *L*. *conchilega* aggregation were found for *Idotea linearis*, *Diogenes pugilator* and *Loligo vulgaris*, while no taxa showed depleted δ^13^C values. In contrast, in spring, *C*. *crangon* showed a depleted δ^13^C value in the aggregation, while no taxa showed enriched δ^13^C values. In Boulogne during autumn, *Nephtys cirrosa* showed a depleted δ^13^C value in the aggregation, while during spring *S*. *rostellatus* showed an enriched δ^13^C value in the aggregation. Regarding δ^15^N, in spring some taxa exhibited enriched values in the *L*. *conchilega* aggregations (*Schistomysis kervillei* and *C*. *crangon* in the BMSM; *Mesopodopsis slabberi* in Boulogne) while *Macoma balthica* showed a depleted value in the *L*. *conchilega* aggregations. In autumn, no taxa with enriched or depleted δ^15^N values in the aggregation were found (Fig 4).

**Table 2:** Stable carbon and nitrogen isotope values (‰, mean ± SD if appropriate) of the primary food sources and consumer taxa of the soft bottom intertidal area of the bay of the Mont Saint-Michel (BMSM) for different combinations of sampling area (*L*. *conchilega* aggregation *vs*. control) and period (spring *vs*. autumn).

		Reef						Control					
		Spring			Autumn			Spring			Autumn		
Taxon		δ^13^C (SD)	δ^15^N (SD)	n	δ^13^C (SD)	δ^15^N (SD)	n	δ^13^C (SD)	δ^15^N (SD)	n	δ^13^C (SD)	δ^15^N (SD)	n
**Osteichthyes**	*Dicentrarchus labrax*	-	-	-	-14.51	15.13	1	-14.12	15.07	1	-	-	-
	*Pleuronectes platessa*	-	-	-	-	-	-	-	-	-	-15.51	13.24	3
	*Platichthys flesus*	-	-	-	-	-	-	-	-	-	-13.99	13.10	1
	*Pomatoschistus* sp.	-15.23 (0.17)	15.64 (0.21)	4	-15.32 (0.14)	14.58 (0.19)	4	-15.29 (0.22)	14.97 (0.28)	4	-15.77 (0.53)	14.62 (0.45)	5
	*Atherina presbyter*	-	-	-	-15.71	14.20	1	-	-	-	-	-	-
	*Solea solea*	-	-	-	-14.67 (0.38)	13.92 (0.17)	7	-	-	-	-	-	-
	*Syngnathus rostellatus*	-	-	-	-	-	-	-18.27 (0.18)	10.89 (0.54)	3	-18.73	10.39	1
**Crustacea**	*Crangon crangon*	-14.38 (0.39)	14.02 (0.11)	4	-13.02 (0.84)	13.30 (0.33)	4	-13.30 (0.31)	13.05 (0.35)	4	-13.14 (1.13)	13.08 (0.70)	4
	*Liocarcinus* sp.	-	-	-	-	-	-	-	-	-	-13.53 (0.58)	13.02 (0.35)	2
	*Carcinus maenas*	-	-	-	-18.17	10.57	1	-	-	-	-	-	-
	*Athanas nitescens*	-18.71	8.45	1	-	-	-	-	-	-	-	-	-
	*Palaemon serratus*	-	-	-	-16.91	13.36	1	-	-	-	-	-	-
	*Portumnus latipes*	-	-	-	-	-	-	-	-	-	-13.70 (0.36)	13.17 (0.38)	2
	*Processa* sp.	-	-	-	-	-	-	-	-	-	-19.13 (0.99)	10.80 (0.87)	2
	*Eualus cranchii*	-	-	-	-	-	-	-	-	-	-18.58 (0.63)	12.23 (0.12)	4
	*Philocheras trispinosus*	-	-	-	-	-	-	-	-	-	-16.19 (0.11)	10.16 (0.18)	2
	*Diogenes pugilator*	-	-	-	-15.59 (0.93)	9.45 (0.19)	3	-	-	-	-16.74 (0.82)	9.74 (0.27)	4
	*Corophium volutator*	-	-	-	-	-	-	-	-	-	-15.76	8.94	1
	*Corophium* sp.	-	-	-	-	-	-	-16.68	5.26	1	-	-	-
	*Gammaru*s sp.	-19.71 (1.48)	7.03 (1.25)	5	-18.99 (1.02)	8.90 (0.61)	4	-20.49 (0.52)	7.33 (1.09)	4	-19.17 (0.58)	8.66 (0.58)	4
	*Abludomelita obtusata*	-19.10	8.89	1	-	-	-	-	-	-	-	-	-
	*Melita* sp.	-19.72	4.93	1	-	-	-	-	-	-	-	-	-
	*Bathyporeia elegans*	-18.32	6.76	1	-	-	-	-	-	-	-	-	-
	*Schistomysis kervillei*	-18.05 (0.45)	12.91 (0.96)	5	-	-	-	-17.59 (0.44)	12.15 (0.91)	4	-	-	-
	*Schistomysis spiritus*	-18.78 (0.81)	9.38 (1.50)	3	-	-	-	-	-	-	-16.61 (0.16)	11.89 (0.37)	3
	*Gastrosaccus spinifer*	-	-	-	-	-	-	-19.76 (0.69)	10.52 (0.80)	3	-18.57 (1.64)	11.16 (0.31)	2
	*Mesopodopsis slabberi*	-	-	-	-	-	-	-	-	-	-17.54 (0.61)	11.94 (0.27)	3
	*Lekanesphaera levii*	-21.22 (2.18)	3.28 (0.62)	3	-	-	-	-	-	-	-17.63 (1.15)	7.77 (0.04)	2
	*Idotea linearis*	-	-	-	-15.49 (0.39)	9.74 (0.28)	3	-19.39	8.48	1	-16.61	9.54	1
	*Idotea balthica*	-	-	-	-17.52 (0.31)	8.81 (0.17)	2	-	-	-	-	-	-
	*Diastylis* sp.	-	-	-	-	-	-	-	-	-	-21.11	9.11	1
**Mollusca**	*Loligo vulgaris*	-	-	-	-16.05 (0.62)	14.89 (0.32)	4	-	-	-	-17.92	15.62	1
	*Buccinum undatum*	-	-	-	-14.85 (0.51)	12.21 (0.05)	3	-14.81	13.38	1	-	-	-
	*Cerastoderma edule*	-18.42	9.37	1	-17.03 (0.15)	9.47 (0.47)	4	-18.50 (0.10)	9.92 (0.36)	4	-	-	-
	*Macoma balthica*	-15.81 (0.16)	9.08 (0.62)	3	-15.32 (0.11)	10.21 (0.48)	4	-15.94 (0.29)	9.91 (0.42)	3	-15.32 (0.11)	9.60 (0.36)	2
**Annelida**	*Lanice conchilega*	-18.55 (0.54)	11.67 (0.57)	4	-17.40 (0.22)	10.97 (0.10)	4	-	-	-	-	-	-
	*Arenicola marina*	-	-	-	-15.94	11.67	1	-	-	-	-	-	-
	*Nephtys cirrosa*	-	-	-	-16.21 (0.29)	11.99 (0.26)	3	-15.88	12.71	1	-16.56 (0.69)	12.71 (0.10)	3
	*Nephtys* sp.	-15.40	11.30	1	-	-	-	-	-	-	-	-	-
	*Nereis* sp.	-	-	-	-15.24	11.24	1	-	-	-	-	-	-
	Polynoinae sp.	-17.31 (0.20)	12.52 (0.31)	3	-	-	-	-	-	-	-	-	-
	*Scoloplos armiger*	-	-	-	-	-	-	-	-	-	-16.25 (1.68)	13.28 (0.16)	2
	Oligochaeta sp.	-	-	-	-	-	-	-21.42	10.09	1	-	-	-
**Cnidaria**	*Rhizostoma pulmo*	-	-	-	-18.25	11.19	1	-	-	-	-	-	-
**Primary sources**	SPOM	-22.66 (0.66)	3.72 (0.38)	3	-20.79 (0.39)	6.30 (0.16)	3	-22.52 (0.26)	4.28 (0.47)	3	-21.10 (0.40)	5.34 (0.23)	3
	MPB_diatom_	-6.84 (0.63)	6.56 (0.25)	4	-11.17 (2.12)	5.77 (1.46)	4	-9.74 (0.36)	6.62 (0.80)	4	-14.08 (4.07)	7.21 (1.60)	3

n = the number of replicates

**Table 3:** Stable carbon and nitrogen isotope values (‰, mean ± SD if appropriate) of the primary food sources and consumer taxa of the soft bottom intertidal area of Boulogne-sur-Mer for different combinations of sampling area (*L*. *conchilega* aggregation *vs*. control) and period (spring *vs*. autumn).

		Reef						Control					
		Spring			Autumn			Spring			Autumn		
Taxon		δ^13^C (SD)	δ^15^N (SD)	n	δ^13^C (SD)	δ^15^N (SD)	n	δ^13^C (SD)	δ^15^N (SD)	n	δ^13^C (SD)	δ^15^N (SD)	n
**Osteichthyes**	*Dicentrarchus labrax*	-	-	-	-16.73 (1.34)	14.69 (0.37)	4	-	-	-	-16.54 (0.05)	15.04 (0.08)	2
	*Pleuronectes platessa*	-24.99	13.47	1	-16.29 (1.09)	12.94 (0.90)	2	-	-	-	-15.96 (0.54)	13.24 (0.41)	7
	Pleuronectidae sp.	-	-	-	-	-	-	-20.95	10.74	1	-	-	-
	Pleuronectidae juv.	-20.48 (1.34)	11.24 (0.91)	4	-	-	-	-	-	-	-	-	-
	Pleuronectidae larvae	-19.77 (0.15)	11.76 (0.34)	4	-	-	-	-	-	-	-	-	-
	*Pomatoschistus microps*	-	-	-	-16.77 (1.63)	14.47 (0.55)	4	-	-	-	-16.90 (0.13)	14.38 (0.32)	3
	*Pomatoschistus* sp.	-17.96 (0.24)	14.89 (0.24)	2	-16.38 (0.13)	14.31 (0.39)	4	-	-	-	-16.76 (0.29)	14.45 (0.25)	10
	*Scopthalmus rhombus*	-	-	-	-17.24	13.18	1	-	-	-	-16.58 (0.67)	13.85 (0.74)	4
	*Echiichthys vipera*	-	-	-	-16.58	15.53	1	-	-	-	-17.31	15.67	1
	*Syngnathus rostellatus*	-17.59	15.74 -	1	-	-	-	-20.00	16.68	1	-	-	-
	*Sprattus sprattus*	-	-	-	-16.48	14.74	1	-	-	-	-	-	-
	Ammodytidae sp.	-	-	-	-	-	-	-18.16 (0.59)	13.96 (0.51)	4	-18.77	14.50	1
**Echinodermata**	*Psammechinus miliaris*	-	-	-	-	-	-	-16.93	11.14	1	-	-	-
**Crustacea**	*Crangon crangon*	-17.13 (2.66)	14.92 (0.73)	4	-15.99 (0.37)	13.50 (0.27)	4	-	-	-	-16.39 (0.22)	13.65 (0.48)	4
	*Crangon crangon* juv.	-	-	-	-	-	-	-17.29 (0.15)	6.74 (0.24)	3	-	-	-
	*Liocarcinus* sp.	-	-	-	-	-	-	-	-	-	-15.35 (2.90)	13.10 (0.21)	3
	*Liocarcinus* sp. juv.	-	-	-	-	-	-	-	-	-	-20.15	10.71	1
	*Carcinus maenas*	-16.05 (0.27)	14.00 (0.26)	3	-16.49 (0.23)	13.88 (0.31)	4	-16.08 (0.12)	14.04 (0.44)	4	-16.62 (0.62)	13.81 (0.43)	5
	*Carcinus maenas* juv.	-14.48	10.26	1	-	-	-	-	-	-	-	-	-
	*Praunus flexuosus*	-	-	-	-	-	-	-	-	-	-18.59	12.80	1
	*Eualus cranchii*	-	-	-	-	-	-	-	-	-	-18.72	13.04	1
	*Gammarus* sp.	-19.48	6.50	1	-	-	-	-	-	-	-21.07	6.66	1
	*Urothoe poseidonis*	-	-	-	-19.66 (1.25)	10.23 (0.32)	4	-	-	-	-	-	-
	*Urothoe* sp. juv.	-	-	-	-19.21 (0.15)	10.31 (0.13)	4	-	-	-	-	-	-
	*Nototropis swammerdamei*	-	-	-	-	-	-	-	-	-	-21.85 (0.97)	7.96 (0.31)	3
	*Schistomysis kervillei*	-	-	-	-	-	-	-16.91	12.31	1	-	-	-
	*Mesopodopsis slabberi*	-16.86 (0.49)	11.12 (0.25)	4	-	-	-	-17.23 (0.58)	10.15 (1.30)	2	-	-	-
	*Gastrosaccus spinifer*	-	-	-	-	-	-	-17.62	11.07	1	-20.78 (1.43)	11.63 (1.36)	3
	*Eurydice pulchra*	-	-	-	-	-	-	-	-	-	-20.70	10.26	1
**Mollusca**	*Venerupis* sp.	-	-	-	-23.29	8.20	1	-	-	-	-	-	-
	*Buccinum undatum*	-	-	-	-	-	-	-	-	-	-15.32	12.80	1
**Annelida**	*Lanice conchilega*	-18.02 (0.68)	10.41 (0.35)	3	-18.99 (0.96)	10.21 (0.25)	4	-	-	-	-	-	-
	*Arenicola marina*	-	-	-	-17.27	13.14	1	-	-	-	-	-	-
	*Nephtys cirrosa*	-	-	-	-17.61 (0.12)	11.88 (0.43)	2	-16.59 (0.40)	11.82 (0.28)	2	-16.33 (0.34)	11.64 (0.10)	3
	*Glycera alba*	-	-	-	-16.89 (0.51)	13.98 (0.95)	2	-	-	-	-	-	-
	*Pholoe minuta*	-17.81	12.39	1	-	-	-	-	-	-	-	-	-
	*Phyllodoce mucosa*	-18.23	12.05	1	-18.10 (0.52)	11.82 (1.09)	2	-	-	-	-	-	-
	Polynoinae sp.	-17.49 (0.48)	12.56 (0.29)	4	-	-	-	-	-	-	-	-	-
	*Lumbrineris* sp.	-	-	-	-19.42	13.14	1	-	-	-	-	-	-
	*Notomastus* sp.	-	-	-	-19.27	10.26	1	-	-	-	-	-	-
**Cnidaria**	Actiniaria sp.	-	-	-	-	-	-	-	-	-	-15.04	13.11	1
**Primary sources**	SPOM	-21.74 (0.22)	4.66 (0.77)	3	-23.24 (0.28)	3.62 (0.66)	3	-21.21 (0.10)	4.10 (1.01)	3	-24.60 (0.70)	3.64 (0.32)	3
	MPB_diatom_	-16.51 (1.83)	2.32 (0.17)	4	-15.38 (2.97)	3.05 (1.06)	4	-12.67 (3.97)	0.91 (0.99)	3	-19.58 (1.18)	1.85 (0.80)	3

n = the number of replicates

### Classical approach towards the effect of *L*. *conchilega* on the food web structure

Variation in the highest trophic position in the food web was small: ranging between 5.47 (control-spring) and 5.63 (*L*. *conchilega* aggregation-spring and control-autumn) in the BMSM and between 4.63 (*L*. *conchilega* aggregation-autumn) and 4.97 (control-spring) in Boulogne (Figs 2 and 3). Similarly, no large differences were observed in the trophic position of consumer taxa which were seasonally co-occurring in the *L*. *conchilega* aggregations and control areas (largest difference: 0.29 TP) (Figs 2 and 3). Following the cluster analysis (and SIMPROF test) of consumers based on their isotope values, the number of trophic groups in a *L*. *conchilega* aggregation area was either equal to (BMSM-autumn and Boulogne-spring) or at least three times as high (BMSM-spring and Boulogne-autumn) as the number of cluster in a control area. The number of functional groups between the *L*. *conchilega* aggregations and control areas was equal, but in the cases where the number of trophic groups in an aggregation were three times higher (*e*.*g*. Fig 2A versus 2B), the functional groups appeared in more trophic groups (Figs 2 and 3; see S1 and S2 Appendices for taxa included in each trophic group).

### Integrated approach towards the effect of *L*. *conchilega* on the food web structure

**Fig 5:**
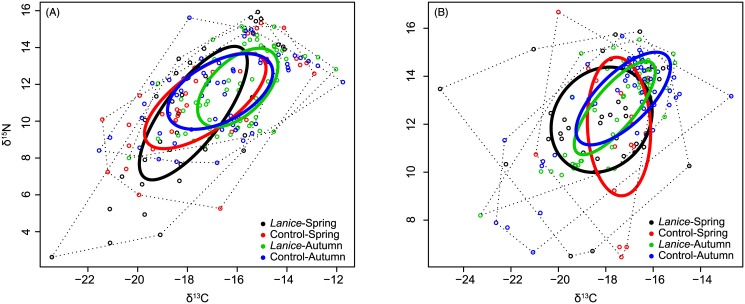
Isotopic niche width of the consumer community. Biplot of δ^13^C and δ^15^N isotope values (‰) of all consumer individuals of the soft bottom intertidal areas of the bay of the Mont Saint-Michel (A) and Boulogne-Sur-Mer (B). Solid lines enclose the standard ellipse area (SEA_C_), representing the isotopic niche of consumer communities for different combinations of sampling area (*L*. *conchilega* aggregation *vs*. control) and period (spring *vs*. autumn). Dotted lines are the convex hulls representing the total niche width of the different consumer communities.

**Fig 6:**
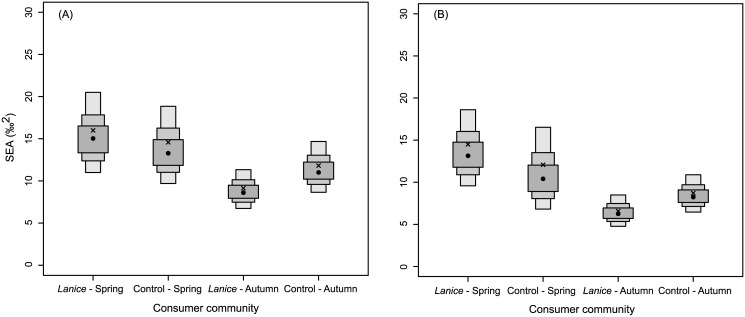
Standard ellipse areas (SEA). Density plots showing the credible intervals of the SEA of consumer communities for different combinations of sampling area (*L*. *conchilega* aggregation *vs*. control) and period (spring *vs*. autumn) in the bay of the Mont Saint-Michel (A) and Boulogne-Sur-Mer (B). Black dots are the mode of the SEA (‰^2^) while the shaded boxes represent the 50% (dark grey), 75% (lighter grey) and 95% (lightest grey) credible intervals. For comparison, small sample size-corrected SEA (SEA_C_) are plotted as crosses.

In general, at both locations the overlap of the standard ellipses for the *L*. *conchilega* aggregations and control areas was high (Fig 5) and found to be higher in spring (BMSM: 11.69‰^2^; Boulogne: 9.05‰^2^) than in autumn (BMSM: 7.83‰^2^; Boulogne: 6.06‰^2^) (Table 4). In spring, the sizes of the standard ellipse areas (SEA_c_) of the *L*. *conchilega* communities was larger than the those of the control communities (BMSM: *L*. *conchilega* aggregation = 15.99‰^2^, control = 14.58‰^2^; Boulogne: *L*. *conchilega* aggregation = 14.50‰^2^, control = 12.08‰^2^) (Fig 6, Table 4). The probability that SEA_B_ of the *L*. *conchilega* aggregation is larger than the SEA_B_ of the control area in spring was 67.33% for the BMSM and 78% for Boulogne (Table 4). On the contrary, in autumn, the sizes of the standard ellipse areas (SEA_c_) of the *L*. *conchilega* communities were smaller than those of the control communities (BMSM: *L*. *conchilega* aggregation = 9.13‰^2^, control = 11.82‰^2^; Boulogne: *L*. *conchilega* aggregation = 6.58‰^2^, control = 8.71‰^2^) (Fig 6, Table 4). The probability that SEA_B_ of the *L*. *conchilega* aggregation is larger than the SEA_B_ of the control area in autumn was 8.74% for the BMSM and 8.38% for Boulogne (Table 4). When comparing the two locations, the SEA_c_ for Boulogne were slightly smaller compared to those of the BMSM (Fig 6).

**Table 4:** Bayesian Layman metrics (NR, CR, CD, MNND and SDNND), small sample size-corrected standard ellipse areas (SEA_C_), and overlap in SEA_C_ (‰2) between pairs of sampling area (*L*. *conchilega* aggregation *vs*. control) and period (spring *vs*. autumn) for the bay of the Mont Saint-Michel (BMSM) and Boulogne-sur-Mer (Boulogne).

										Reef		Control	
Location	Sampling area	Period	n	NR	CR	CD	MNND	SDNND	SEAc (‰^2^)	Spring	Autumn	Spring	Autumn
BMSM	Reef	Spring	40	13.62	9.25	3.78	1.62	1.29	15.99	-	4.70	11.69	8.64
		Autumn	57	9.66	9.68	2.85	1.14	1.22	9.13	0.996	-	6.98	7.83
	Control	Spring	35	11.81	10.38	3.70	1.78	1.46	14.58	0.673	0.017	-	10.88
		Autumn	36	10.69	11.52	3.14	1.17	1.18	11.82	0.924	0.087	0.812	-
Boulogne	Reef	Spring	35	12.00	12.31	3.51	1.81	1.80	14.50	-	5.76	9.05	6.37
		Autumn	47	10.19	10.54	2.85	1.15	1.56	6.58	1.000	-	5.04	6.06
	Control	Spring	20	11.10	7.68	3.20	2.07	1.71	12.08	0.780	0.020	-	5.79
		Autumn	56	12.11	11.21	3.25	1.27	1.53	8.71	0.987	0.084	0.846	-

The upper parts of the matrices show the overlap in SEAC between pairs, while the lower parts show the Bayesian probability that the SEA of group 1 is smaller than the SEA of group 2. NR = δ^15^N range, CR = δ^13^C range, CD = mean distance to centroid, MNND = mean nearest neighbour distance, SDNND = standard deviation of the nearest neighbour distance. n = the number of individuals used to calculate the metrics.

**Fig 7:**
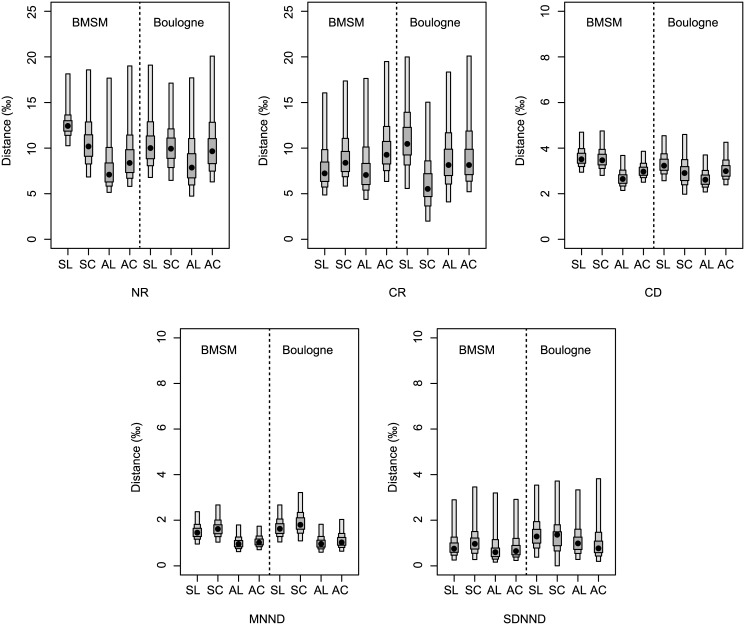
Bayesian Layman metrics. Density plot showing the uncertainty of the Bayesian Layman metrics (NR = δ^15^N range, CR = δ^13^C range, CD = mean distance to centroid, MNND = mean nearest neighbour distance, SDNND = standard deviation of the nearest neighbour distance) for different combinations of location (BMSM *vs*. Boulogne), sampling area (*L*. *conchilega* aggregation *vs*. control) and period (spring *vs*. autumn). Black dots represent the modes, while the shaded boxes represent the 50% (dark grey), 75% (light grey) and 95% (white) credible intervals. Note the different scales of distance (‰) for NR and CR *vs*. CD, MNND and SDNND. SL = spring-*L*. *conchilega* aggregation; SC = spring-control; AL = autumn-*L*. *conchilega* aggregation; AC = autumn-control.

Visual analysis of the credible intervals of the Bayesian implementation of the Layman metrics showed for all 8 food webs a high overlap in the *δ* ^*15*^ *N range* (NR), the *δ* ^*13*^ *C range* (CR) and the *standard deviation of the nearest neighbour distance* (SDNND) (Fig 7, Table 4). Credible intervals of the *mean distance to centroid* (CD) and the *mean nearest neighbour distance* (MNND) overlapped largely between *L*. *conchilega* and control communities, while slightly lower overlaps between spring and autumn communities were noted (Fig 7, Table 4).

## Discussion

Notwithstanding the engineering effects of *L*. *conchilega* on the physical characteristics of the environment [13, 38] and the subsequent alteration in the abundance and biomass of the primary producers and a broad spectrum of macrofaunal organisms [*e*.*g*. 17, 20], the current study shows that the ecosystem engineering effect of *L*. *conchilega* does not directly affect the structure and isotopic niche of the food web of a soft-bottom intertidal ecosystem.

### Primary food sources

Primary food sources largely determine the food web structure by fuelling higher trophic levels in the system. Bulk organic matter (SPOM) and benthic diatoms (MPB_diatom_) are the main food sources of the soft-bottom intertidal community, both in the presence and absence of *L*. *conchilega*. The tubes of *L*. *conchilega*, and animal tubes in soft bottom marine environments in general, can perturb the local flow conditions [*e*.*g*. 39], promoting a higher deposition rate of detrital organic matter (De Smet *et al*. unpublished) and an increased microbial colonisation, with bacteria considered as an important food source of deposit-feeding fauna [40]. The δ^13^C values of SPOM measured in this study (-22.23 ± 1.28‰) are in the same range as δ^13^C values of pure phytoplankton in temperate coastal areas and estuaries [*e*.*g*. 41, 42, 43]. MPB_diatom_ on its turn is more enriched in ^13^C (-13.14 ± 4.39‰) compared to SPOM, which is in line with the general trend that benthic algae in coastal environments have higher δ^13^C values compared to phytoplankton [44]. Therefore, we suggest that the sampled SPOM is a mixture containing a rather small amount of locally produced MPB_diatom_ and predominantly water column-derived suspended POM. Stable isotope values of the primary food sources were shown to be largely similar between the *L*. *conchilega* aggregations and control areas, implying an unaltered diversity of the most dominant primary resources in the presence of the tubeworm. Hence, the ecosystem engineering activity of *L*. *conchilega* does not directly modify the base of the food web. Isotope values differ rather seasonally: the δ^13^C value of MPB_diatom_ in the BMSM is higher in spring than in autumn; however the opposite is the case for SPOM. Since changes in the amount of benthic diatoms among seasons are small [16] and because they most probably only form a minor fraction of the bulk organic matter, its isotope signature seems to be masked by the high quantities of depleted POM in spring [16]. Conversely, in autumn the amounts of depleted POM are much lower [16] leading to enriched benthic diatom-dominated bulk organic matter. Because SPOM is the most important carbon source for primary consumers in this study, the soft-bottom intertidal food web seems to be mainly driven by carbon input from the water column, rather than by *in situ* primary production by benthic diatoms. This finding confirms the important trophic contribution of near shore phytoplankton to sandy beach macrofauna [45, 46].

### Consumers

Independent of the location, isotope values of primary food sources do not differ greatly among sampling areas and most consumer taxa co-occurring at both sampling areas exhibit similar isotope values, indicating that consumer taxa generally do not shift their diet when feeding in a *L*. *conchilega* aggregation. Moreover, the largely unchanged consumer diets are reflected in the almost invariable ranges in δ^15^N and δ^13^C and the highly similar trophic positions and isotopic niche widths of consumer communities of *L*. *conchilega* aggregations and control areas. Nonetheless, the high probability (at least 67%) that the isotopic niche width of the *L*. *conchilega* communities is larger than the bare sand communities in spring, in combination with the deviating isotope values of some consumer taxa in the *L*. *conchilega* aggregations implies that some consumers do shift their diet preference depending on the sampling area and the period. A diet shift is for instance the case for the brown shrimp *C*. *crangon*, which is one of the most abundant species in a *L*. *conchilega* aggregation [16]. When feeding in the *L*. *conchilega* aggregation in the BMSM during spring, *C*. *crangon* has a depleted δ^13^C value and an enriched δ^15^N value compared to a bare sand plot. Deviating isotope values of taxa in the *L*. *conchilega* aggregation can be the result of the uptake of a specific food source or of the different community composition in the aggregations. Firstly, the specific food uptake can be the case if a consumer feeds on a more δ^13^C enriched or depleted prey source which is merely available or more readily accessible in a *L*. *conchilega* aggregation due to the aggregation’s specific habitat characteristics (*e*.*g*. shelter provision; [47]). A prerequisite for this kind of change in the isotope values is that the consumer can circumvent the tidal cycle and remain in the *L*. *conchilega* aggregation for a longer period of time or that the consumer is able to find its way back to the aggregation. The former is likely for macrobenthic animals and consumer taxa showing burying behaviour when the water retreats such as *C*. *crangon*, (pers. obs. in experimental setups, [48]). More mobile, non-burrowing epifauna with deviating isotope values in the *L*. *conchilega* aggregation, such as *L*. *vulgaris* and *S*. *rostellatus*, are believed to be able to reoccupy their position in the aggregation at high tide. Secondly, the different composition of the associated fauna might affect the food selectivity of consumers. The higher number of trophic groups in the *L*. *conchilega* aggregation suggests a slightly different food selectivity and hence a decrease in the competition between consumers which is supposed to be beneficial in the densely populated *L*. *conchilega* aggregations. Despite the reported locally increased species richness in a *L*. *conchilega* aggregation [47, 49], the number of taxa between the *L*. *conchilega* and control areas was not different in this study. Nonetheless, standardised sampling techniques were used consistently throughout this study and we believe that the gathered data veraciously reflect the observed food web structure.

While most taxa do not show a diet shift in the *L*. *conchilega* aggregations, a more in-depth view reveals that there might be an indirect engineering effect of *L*. *conchilega* on a minor fraction of the consumer taxa owing to a specific food uptake and/or the different community composition in the aggregations.

Isotopic niche width was not different among sampling areas (*L*. *conchilega* aggregation *vs*. control), but differences among periods (spring *vs*. autumn) were shown to be slightly larger, indicating that the isotopic niche width of the consumer communities among periods is less alike than the isotopic niche width of the communities among sampling areas. A shift in the diet of the consumer taxa from spring to autumn and vice versa most reasonably explains the observed differences [50]. Species packing and hence trophic redundancy among sampling areas (as measured by MNND and SDNND metrics) seems not to be affected, pointing to an unaltered stability of the food web in the presence of *L*. *conchilega*. However, based on the increased sediment stability in the presence of *L*. *conchilega* [13, 51], a stabilizing effect of the ecosystem engineer on the food web base was expected. The observed unaltered stability can be related to the dependence of the food web on water column-derived primary production (SPOM) rather than *in situ* primary production from the sediment. Comparison of trophic redundancy and overall species packing among periods reveals that the autumn communities showed an increase [by the lower values of MNND and to some extent by the lower values of CD; 52], compared to their counterparts in spring. Hence, food web stability is slightly higher in autumn compared to spring.

Following the largely invariable range of δ^15^N, the trophic position of consumers in the food web seems not to be affected by the presence of the *L*. *conchilega* aggregations. It should be noted however that the estimates of the TP in this study are much higher than the trophic levels of the different compartments of other intertidal food webs based on phytoplankton and detritus [53]. The slightly higher TPs in the BMSM compared to Boulogne most probably result from the considerable variation in the δ^15^N of the location-specific trophic baseline. The selection of an appropriate baseline is a crucial step and will largely influence the estimation of a consumer’s TP [33, 34]. Therefore, the inclusion of smaller-sized benthos (meiofauna; mainly nematodes and harpacticoid copepods), as a trophic linkage to macrofauna and an important structural component of the benthic community [54], is recommended for future research. Although the primary consumers selected as a baseline in this study are not optimal, it does not conflict with our main interest: the TP of a consumer taxon in a *L*. *conchilega* aggregation relative to a control area.

### Linking ecosystem engineering and food webs

Although integrated studies on ecosystem engineering and food web structure are rare in marine research, the outcomes of our study are largely in line with existing knowledge. Rigolet *et al*. [7] investigated changes in the benthic food web structure of an *Amphiura filiformis* habitat colonised by the engineering tubicolous amphipod *Haploops nirae*. Despite altering local sediment features and positively affecting the local biodiversity and associated species assemblages [55], *H*. *nirae* did not affect the food web structure as based on ranges in δ^13^C and δ^15^N. Similarly, Baeta *et al*. [56] found neither differences in the planktonic nor the benthic food web structure between a site dominated by eelgrass *Zostera noltei* and bare sediment. A study by Botto *et al*. [57] shows that the burrowing crab *Neohelice granulata*, an engineer in SW Atlantic coastal areas, modifies δ^15^N values of sediments and primary producers by 3 to 7‰. Some consumers associated with the sedimentary environment reflected the enriched N values, but the overall food web structure in areas with and without the crab however remained largely unaltered.

Apart from the well-documented effects of an ecosystem engineer on its environment and on the composition of a community, the results of the current and previous studies did not show a global impact of the presence of ecosystem engineers on the marine food web structure; contrasting recently formulated hypotheses [4]. As opposed to the strong impact of *L*. *conchilega* on the benthic assemblage, the ecosystem engineer’s influence on the water column is probably too limited to substantially stir the global structure of the soft-bottom intertidal food web, which is mainly driven by water-column derived primary production.

## Supporting Information

S1 Appendix Mean stable isotope values of the consumer clusters of the bay of the Mont Saint-Michel (BMSM). Classification of consumer taxa with similar food uptake (δ^13^C) and trophic level (δ^15^N) for different combinations of sampling area and period in the BMSM, based on agglomerative hierarchical cluster analyses and similarity profile (SIMPROF) permutation tests. Cluster names match the clusters defined in the δ^13^C — δ^15^N biplot of the BMSM (Fig 2). For each of the clusters, the mean δ^13^C and δ^15^N values (±SD) are displayed, as well as the taxonomic composition and the number of replicates per taxon (n).
(DOCX)

S2 Appendix Mean stable isotope values of the consumer clusters of the beach of Boulogne-sur-Mer. Classification of consumer taxa with similar food uptake (δ^13^C) and trophic level (δ^15^N) for different combinations of sampling area and period in Boulogne-sur-Mer, based on agglomerative hierarchical cluster analyses and similarity profile (SIMPROF) permutation tests. Cluster names match the clusters defined in the δ^13^C — δ^15^N biplot of Boulogne (Fig 3). For each of the clusters, the mean δ^13^C and δ^15^N values (±SD) are displayed, as well as the taxonomic composition and the number of replicates per taxon (n).
(DOCX)
