# Supplementary material for: Integrating Ecosystem Engineering and Food Web Ecology: Testing the Effect of Biogenic Reefs on the Food Web of a Soft-Bottom Intertidal Area
Source: PLoS One. 2015 Oct 23;10(10):e0140857. doi: 10.1371/journal.pone.0140857 (PMC4619716; doi:10.1371/journal.pone.0140857)
Supplement: S1 Appendix — Classification of consumer taxa with similar food uptake (δ13C) and trophic level (δ15N) for different combinations of sampling area and period in the BMSM, based on agglomerative hierarchical cluster analyses and similarity profile (SIMPROF) permutation tests. Cluster names match the clusters defined in the δ13C — δ15N biplot of the BMSM (Fig 2). For each of the clusters, the mean δ13C and δ15N values (±SD) are displayed, as well as the taxonomic composition and the number of replicates per taxon (n). (DOCX) [file pone.0140857.s001.docx]

| Spring | | | | | | | | | | |
| --- | --- | --- | --- | --- | --- | --- | --- | --- | --- | --- |
| *L. conchilega* aggregation | | | | |  | Control | | | | |
| Cluster | δ^13^C ± SD | δ^15^N ± SD | Species | n |  | Cluster | δ^13^C ± SD | δ^15^N ± SD | Species | n |
| 1A | -15.23 ± 0.17 | 15.64 ± 0.21 | *Pomatoschistus* sp. | 4 |  | 1B | -14.47 ± 1.04 | 13.93 ± 1.06 | *Buccinum undatum* | 1 |
| 2A | -14.38 ± 0.39 | 14.02 ± 0.11 | *Crangon crangon* | 4 |  |  |  |  | *Crangon crangon* | 4 |
| 3A | -17.74 ± 0.50 | 12.88 ± 0.59 | *Schistomysis kervillei* | 4 |  |  |  |  | *Dicentrarchus labrax* | 1 |
|  |  |  | *Lanice conchilega* | 1 |  |  |  |  | *Pomatoschistus* sp. | 4 |
|  |  |  | Polynoinae sp. | 3 |  |  |  |  | *Nepthys cirrosa* | 1 |
| 4A | -15.71 ± 0.24 | 9.64 ± 1.22 | *Macoma balthica* | 3 |  | 2B | -18.57 ± 1.60 | 9.81 ± 1.90 | Corophium sp. | 1 |
|  |  |  | *Nephty*s sp. | 1 |  |  |  |  | *Gammarus* sp. | 4 |
| 5A | 18.75 ± 0.59 | 11.03 ± 0.68 | *Schistomysis kervillei* | 1 |  |  |  |  | *Gastrosaccus spinifer* | 3 |
|  |  |  | *Schistomysis spiritus* | 2 |  |  |  |  | *Idotea linearis* | 1 |
|  |  |  | *Lanice conchilega* | 3 |  |  |  |  | *Schistomysis kervillei* | 4 |
| 6A | -19.33 ± 1.16 | 6.80 ± 1.94 | *Athanas nitescens* | 1 |  |  |  |  | *Syngnathus rostellatus* | 3 |
|  |  |  | *Gammarus* sp. | 5 |  |  |  |  | *Cerastoderma edule* | 4 |
|  |  |  | *Lekanesphaera levii* | 2 |  |  |  |  | *Macoma balthica* | 3 |
|  |  |  | *Abludomelita obtusata* | 1 |  |  |  |  | Oligochaeta sp. | 1 |
|  |  |  | *Melit*a sp. | 1 |  |  |  |  |  |  |
|  |  |  | *Schistomysis spiritus* | 1 |  |  |  |  |  |  |
|  |  |  | *Bathyporeia elegans* | 1 |  |  |  |  |  |  |
|  |  |  | *Cerastoderma edule* | 1 |  |  |  |  |  |  |
| 7A | -23.45 | 2.62 | *Lekanesphaera levii* | 1 |  |  |  |  |  |  |
| Autumn | | | | | | | | | | |
| *L. conchilega* aggregation | | | | |  | Control | | | | |
| Cluster | δ^13^C ± SD | δ^15^N ± SD | Species | n |  | Cluster | δ^13^C ± SD | δ^15^N ± SD | Species | n |
| 1C | -15.53 ± 0.25 | 14.71 ± 0.34 | *Atherina presbyter* | 1 |  | 1D | -17.92 | 15.62 | *Loligo vulgaris* | 1 |
|  |  |  | *Loligo vulgaris* | 3 |  | 2D | -15.54 ± 0.17 | 14.81 ± 0.16 | *Pomatoschistus* sp. | 4 |
|  |  |  | *Pomatoschistus* sp. | 4 |  | 3D | -14.80 ± 0.34 | 13.33 ± 0.20 | *Crangon crangon* | 1 |
| 2C | -16.95 ± 0.04 | 13.89 ± 0.75 | *Palaemon serratus* | 1 |  |  |  |  | *Pleuronectes platessa* | 2 |
|  |  |  | *Loligo vulgaris* | 1 |  |  |  |  | *Scoloplos armiger* | 1 |
| 3C | -14.85 ± 0.39 | 13.99 ± 0.49 | *Crangon crangon* | 1 |  | 4D | -13.56 ± 0.47 | 13.15 ± 0.29 | *Crangon crangon* | 2 |
|  |  |  | *Dicentrarchus labrax* | 1 |  |  |  |  | *Liocarcinus* sp. | 2 |
|  |  |  | *Solea solea* | 7 |  |  |  |  | *Platichthys flesus* | 1 |
| 4C | -12.68 ± 0.62 | 13.27 ± 0.40 | *Crangon crangon* | 3 |  |  |  |  | *Portumnus latipes* | 2 |
| 5C | -16.48 ± 1.25 | 10.48 ± 1.14 | *Buccinum undatum* | 3 |  | 5D | -11.75 | 12.12 | *Crangon crangon* | 1 |
|  |  |  | *Carcinus maenas* | 1 |  | 6D | -17.65 ± 1.54 | 10.72 ± 1.70 | *Diogenes pugilator* | 4 |
|  |  |  | *Diogenes pugilator* | 3 |  |  |  |  | *Pleuronectes platessa* | 1 |
|  |  |  | *Rhizostoma pulmo* | 1 |  |  |  |  | *Pomatoschistu*s sp. | 1 |
|  |  |  | *Gammarus* sp. | 2 |  |  |  |  | *Corophium volutator* | 1 |
|  |  |  | *Idotea balthica* | 2 |  |  |  |  | *Diastyli*s sp. | 1 |
|  |  |  | *Idotea linearis* | 3 |  |  |  |  | *Gammarus* sp. | 4 |
|  |  |  | *Arenicola marina* | 1 |  |  |  |  | *Gastrosaccus spinifer* | 2 |
|  |  |  | *Cerastoderma edule* | 4 |  |  |  |  | *Idotea linearis* | 1 |
|  |  |  | *Lanice conchilega* | 4 |  |  |  |  | *Lekanesphaera levii* | 2 |
|  |  |  | *Macoma balthica* | 4 |  |  |  |  | *Mesopodopsis slabberi* | 3 |
|  |  |  | *Nephtys cirrosa* | 3 |  |  |  |  | *Philocheras trispinosus* | 2 |
|  |  |  | *Nereis* sp. | 1 |  |  |  |  | *Processa* sp. | 2 |
| 6C | -19.76 ± 0.74 | 8.52 ± 0.74 | *Gammarus* sp. | 2 |  |  |  |  | *Schistomysis spiritus* | 3 |
|  |  |  |  |  |  |  |  |  | *Syngnathus rostellatus* | 1 |
|  |  |  |  |  |  |  |  |  | *Eualus cranchii* | 4 |
|  |  |  |  |  |  |  |  |  | *Macoma balthica* | 2 |
|  |  |  |  |  |  |  |  |  | *Nephtys cirrosa* | 3 |
|  |  |  |  |  |  |  |  |  | *Scoloplos armiger* | 1 |
