# Supplementary material for: Integrating Ecosystem Engineering and Food Web Ecology: Testing the Effect of Biogenic Reefs on the Food Web of a Soft-Bottom Intertidal Area
Source: PLoS One. 2015 Oct 23;10(10):e0140857. doi: 10.1371/journal.pone.0140857 (PMC4619716; doi:10.1371/journal.pone.0140857)
Supplement: S2 Appendix — Classification of consumer taxa with similar food uptake (δ13C) and trophic level (δ15N) for different combinations of sampling area and period in Boulogne-sur-Mer, based on agglomerative hierarchical cluster analyses and similarity profile (SIMPROF) permutation tests. Cluster names match the clusters defined in the δ13C — δ15N biplot of Boulogne (Fig 3). For each of the clusters, the mean δ13C and δ15N values (±SD) are displayed, as well as the taxonomic composition and the number of replicates per taxon (n). (DOCX) [file pone.0140857.s002.docx]

| Spring | | | | | | | | | | |
| --- | --- | --- | --- | --- | --- | --- | --- | --- | --- | --- |
| *L. conchilega* aggregation | | | | |  | Control | | | | |
| Cluster | δ^13^C ± SD | δ^15^N ± SD | Species | n |  | Cluster | δ^13^C ± SD | δ^15^N ± SD | Species | n |
| 1E | -18.18 ± 2.10 | 12.19 ± 2.16 | *Carcinus maenas* | 3 |  | 1F | -17.44 ± 1.30 | 11.90 ± 2.81 | Ammodytidae sp. | 4 |
|  |  |  | *Carcinus maenas* juv. | 1 |  |  |  |  | *Carcinus maenas* | 4 |
|  |  |  | *Crangon crangon* | 4 |  |  |  |  | *Psammechinus miliaris* | 1 |
|  |  |  | *Pleuronectes platessa* | 1 |  |  |  |  | *Crangon crangon* juv. | 3 |
|  |  |  | *Pomatoschistus* sp. | 2 |  |  |  |  | *Gastrosaccus spinifer* | 1 |
|  |  |  | *Gammarus* sp. | 1 |  |  |  |  | *Mesopodopsis slabberi* | 2 |
|  |  |  | *Mesopodopsis slabberi* | 4 |  |  |  |  | Pleuronectidae sp. | 1 |
|  |  |  | Pleuronectidae juv. | 4 |  |  |  |  | *Schistomysis kervillei* | 1 |
|  |  |  | Pleuronectidae larvae | 4 |  |  |  |  | *Syngnathus rostellatus* | 1 |
|  |  |  | *Syngnathus rostellatus* | 1 |  |  |  |  | *Nepthys cirrosa* | 2 |
|  |  |  | *Lanice conchilega* | 3 |  |  |  |  |  |  |
|  |  |  | *Pholoe minuta* | 1 |  |  |  |  |  |  |
|  |  |  | *Phyllodoce mucosa* | 1 |  |  |  |  |  |  |
|  |  |  | Polynoinae sp. | 4 |  |  |  |  |  |  |
| Autumn |  |  |  |  |  |  |  |  |  |  |
| *L. conchilega* aggregation | | | | |  | Control | | | | |
| Cluster | δ^13^C ± SD | δ^15^N ± SD | Species | n |  | Cluster | δ^13^C ± SD | δ^15^N ± SD | Species | n |
| 1G | -16.71 ± 1.01 | 13.99 ± 0.78 | *Carcinus maenas* | 4 |  | 1H | -16.59 ± 1.10 | 13.72 ± 0.90 | Actiniaria sp. | 1 |
|  |  |  | *Crangon crangon* | 4 |  |  |  |  | Ammodytidae sp. | 1 |
|  |  |  | *Dicentrarchus labrax* | 4 |  |  |  |  | *Buccinum undatum* | 1 |
|  |  |  | *Echiichthys vipera* | 1 |  |  |  |  | *Carcinus maenas* | 5 |
|  |  |  | *Pleuronectes platessa* | 2 |  |  |  |  | *Crangon crangon* | 4 |
|  |  |  | *Pomatoschistus microps* | 4 |  |  |  |  | *Dicentrarchus labrax* | 2 |
|  |  |  | *Pomatoschistus* sp. | 4 |  |  |  |  | *Echiichthys vipera* | 1 |
|  |  |  | *Scopthalmus rhombus* | 1 |  |  |  |  | *Liocarcinus* sp. | 3 |
|  |  |  | *Sprattus sprattus* | 1 |  |  |  |  | *Pleuronectes platessa* | 7 |
|  |  |  | *Arenicola marina* | 1 |  |  |  |  | *Pomatoschistus microps* | 3 |
|  |  |  | *Glycera alba* | 2 |  |  |  |  | *Pomatoschistus* sp. | 10 |
|  |  |  | *Lumbrineris* sp. | 1 |  |  |  |  | *Scopthalmus rhombus* | 4 |
|  |  |  | *Phyllodoce mucosa* | 1 |  |  |  |  | *Gastrosaccus spinifer* | 1 |
| 2G | -17.70 ± 0.13 | 11.02 ± 0.89 | *Lanice conchilega* | 1 |  |  |  |  | *Praunus flexuosus* | 1 |
|  |  |  | *Nephtys cirrosa* | 2 |  |  |  |  | *Eualus cranchii* | 1 |
|  |  |  | *Phyllodoce mucosa* | 1 |  |  |  |  | *Nephtys cirrosa* | 3 |
|  |  |  | *Urothoe poseidonis* | 1 |  | 2H | -21.30 ± 0.92 | 9.16 ± 1.72 | *Liocarcinus* sp. juv. | 1 |
| 3G | -20.30 ± 0.44 | 9.98 ± 0.09 | *Lanice conchilega* | 1 |  |  |  |  | *Gammarus* sp. | 1 |
|  |  |  | *Urothoe poseidonis* | 2 |  |  |  |  | *Gastrosaccus spinifer* | 2 |
| 4G | -19.23 ± 0.15 | 10.33 ± 0.11 | *Lanice conchilega* | 2 |  |  |  |  | *Nototropis swammerdamei* | 3 |
|  |  |  | *Notomastus* sp. | 1 |  |  |  |  | *Eurydice pulchra* | 1 |
|  |  |  | *Urothoe* sp. juv. | 4 |  |  |  |  |  |  |
| 5G | -19.72 | 10.71 | *Urothoe poseidonis* | 1 |  |  |  |  |  |  |
| 6G | -23.29 | 8.2 | *Venerupis* sp. | 1 |  |  |  |  |  |  |
